# How theory and design-based research can mature PBL practice and research

**DOI:** 10.1007/s10459-019-09940-2

**Published:** 2019-11-13

**Authors:** Diana H. J. M. Dolmans

**Affiliations:** Maastricht University, School of Health Professions Education (SHE), Maastricht, The Netherlands

**Keywords:** Problem-based learning, Design-based research, Learning theories

## Abstract

Many educational institutions in higher education switched to problem-based learning (PBL) in the last 5 decades. Despite its’ successful implementation worldwide, many institutions still encounter problems in their daily teaching practices that limit deep learning in students. This raises the question: How else can we look at PBL practice and research? The main argument of this reflective paper is to better align PBL practice with the theories or principles of contextual, constructive, self-directed and collaborative learning. This paper explains what these principles or theories are. In addition, it discusses a new way to bridge theory and practice: design-based research (DBR), which combines redesigning theory-based teaching practices with investigating these practices in close collaboration with various stakeholders. There is no one-size-fits-all solution to address the problems encountered in PBL. We should be very careful in drawing conclusions about which PBL approach works best. No single solution works optimally under all conditions. At most, DBR can help us gain better insight into why PBL with certain characteristics, preferably based on theory, might work in a specific context with particular goals in mind.

## Introduction

Higher education institutes all over the world have implemented problem-based learning (PBL) in recent years in order to stimulate students to take a deep approach to learning. In PBL, small groups (6–10 students) discuss problems, led by a teacher. The teacher starts the discussion by activating the students’ prior knowledge of the topic, without allowing for any preparation or self-study. After the initial discussion, questions are formulated by students that need further study. Following individual self-study, the group meets again to synthesize and discuss what they have learned. In addition, a limited number of lectures and skills trainings are offered to the students to support their learning in the small groups. In PBL, students are expected to activate their prior knowledge, to relate new information to their prior knowledge, to structure ideas, to look for underlying principles and critically evaluate their knowledge. In other words, the idea behind PBL is to get students to adopt a deep approach to studying.

Although PBL is implemented worldwide, many schools struggle with PBL in practice for various reasons; e.g. because PBL does not always result in deep learning (Dolmans et al. 2016). Problems encountered with PBL have been claimed to stem from poor implementation, resulting in a program inconsistent with insights into learning (Dolmans et al. 2005). If PBL is not implemented well because, e.g., it relies on poor quality PBL problems or large groups, deep processing will be hard to achieve.

In this reflective paper we argue that we should adapt the practice of PBL to align it better with theories. We should look at PBL from the perspective of theory. In addition, we need to investigate the redesigned PBL practices and conduct studies that will both inform practice and advance our understanding of how certain characteristics or ingredients of PBL might work to enhance deep learning.

This paper contains three main parts. Part I explains how theories or principles can be used to inform PBL practice. Part II gives examples of how to redesign PBL in practice, resulting in theory-based adaptations. Part III argues how and why we should embrace design-based research (DBR) to mature PBL practice and develop and test theories. This reflective paper ends with a conclusion and discussion section.

## Part I: Theories and principles

PBL has been claimed to have the potential to prepare students effectively for future learning in that it fits well with four principles: contextual, constructive, self-directed and collaborative learning (Dolmans et al. 2005). But, what do these principles or theories actually mean?

First of all, what is meant with the word theory? A theory can be considered as a way of thinking about how something might work, as Bordage (2009) explains. A theory often consists of a set of principles to define or analyze a problem from a particular perspective. A theory might help us understand a certain problem and offer solutions to address the problem from a particular perspective. Usually a set of theories is needed to understand and solve a problem (Bordage 2009; Rees and Monrouxe 2010).

### Contextual learning

The contextual learning principle implies that learning preferably starts by engaging students in tasks derived from a professionally relevant context in order to stimulate transfer of learning to new situations. In other words, center all learning around whole, complex, ill-defined tasks, so called task-centered instruction (Francom 2016). In order to prepare learners for future learning in which they can deal with a variety of new tasks or problems, we should not only expose learners to new clarifying information, e.g. by means of lectures, but preferably also stimulate them to compare and contrast tasks derived from relevant contexts (Bransford and Schwartz 1999). Through discussing contrasting tasks, learners notice what critical features are of a variety of tasks. This is especially true for ill-defined tasks, i.e. tasks with one or more unknown elements and a variety of solutions which are often situated in the real world (Jonassen 1997). When designing or redesigning curricula, meaningful, authentic or professionally relevant tasks should be the starting point (Harden 2018). Learning tasks can be presented as problems, but also as cases or projects. The basic idea is that learning tasks are aimed at enabling the student to acquire an integrated set of knowledge, skills and attitudes, instead of a fragmented set of specific knowledge, skills and attitudes. Instead of teaching knowledge, skills and attitudes piece by piece, the tasks set should enhance the integration of knowledge, skills and attitudes. In addition, teachers should offer a variety of tasks, ordered from the simple to the complex, and provide the learner with scaffolds that diminish at each level of complexity to enhance the transfer of knowledge to new situations (Merrill 2002, 2012; Van Merriënboer and Kirschner 2017). In PBL, students’ learning starts from discussing problems relevant for the students’ future profession. Problems are at the centre of learning and are based on real-life problems encountered in the future work setting.

### Constructive learning

PBL should also stimulate students to acquire new knowledge, preferably in an active process in which they construct and reconstruct their knowledge networks and create meaning to gain a deep understanding. This can be done by encouraging students to activate their prior knowledge, because prior knowledge is the foundation for acquiring new knowledge, according to the prior knowledge activation theory (Schmidt 1993; Norman and Schmidt 2000). It can also be done by encouraging students to elaborate (Schmidt 1993). According to the elaboration theory, learners will usually start with a wide-angle view showing the major parts of a problem, which lacks much detail, after which the learner will zoom in on a certain part. Thereafter the learner needs to zoom back out to see the relationships among the different parts of the problem to enhance meaningful learning and avoid segmented information (Reigeluth et al. 1980). In other words, elaboration is about relating new information with prior knowledge. Stimulating students to generate meaningful new relations between prior knowledge and new knowledge positively affects long-term memory (Van Blankenstein et al. 2011). In their influential papers reflecting on the available evidence for the theoretical claims made about PBL, Norman and Schmidt (1992) and Schmidt (1993) described the benefits to long-term recall derived from the activation, elaboration and self-explanation of prior knowledge. Other authors as well, such as Dunlosky et al. (2013), conclude from a comparison of effective learning strategies that elaboration and self-explanation show enough promise to recommend their use. In general, students in PBL are recommended to actively construct their knowledge network and adopt a deep approach to study in which they relate new ideas to their prior knowledge and elaborate, give meaning and critically evaluate what they learn (Biggs et al. 2001; Dolmans et al. 2016). The initial discussion in the group and the final discussion in the group are aimed at stimulating students to construct and reconstruct their knowledge network. During the discussion, students are encouraged to ask critical questions, to reason, to give explanations to others, to discuss cognitive disagreements, and to apply new knowledge to varied tasks and real life problems.

### Self-directed learning

The idea behind the theory of self-directed learning (SDL) is that students take the initiative to determine their own learning needs, set goals and strategies to achieve these goals and evaluate their learning (Knowles 1975). SDL theory postulates not only that learners should take the lead in their learning but also that the learning environment should be designed to encourage the learner to take this lead by giving autonomy and providing opportunities for students to customize their learning to their individual learning needs. The concept of SDL emphasizes that the learning environment should be designed to foster self-directed learning; e.g. by stimulating students to formulate questions or define learning issues that need further study and select relevant resources they need to study to gain a better understanding (Loyens et al. 2008). This is not meant as a plea to limit the support given to the learner. Students should simultaneously be offered both plenty of support in the learning environment and the option to self-direct their learning (Kusurkar and Croiset 2015). Given that students after the initial discussion in the group diagnose their own learning needs, generate questions or list learning issues that need further study, PBL is assumed to stimulate students to self-direct their learning. Instead of SDL, often the term self-regulated learning (SRL) is used. SRL is about students regulating, i.e. monitoring and controlling their study behavior or learning, but also about regulating feelings, emotions and motivation (Boekaerts 1997; Pintrich 2000; Zimmerman 1990). Monitoring implies that a learner has meta-cognitive awareness of what he already knows e.g. by regularly evaluating his performance. Controlling implies that a learner adapts his learning to his needs. For example, while studying materials related to the learning issues, a student should evaluate his understanding, or test himself and use another resource if he feels the text is difficult to understand. Both processes of monitoring and control are strongly related and have a positive relationship with learning (Zimmerman 1990), although there is also some evidence that it might vary across contexts which strategy is effective (Sitzmann and Ely 2011; Dunlosky et al. 2013). There are many similarities between SDL and SRL in terms of goal-setting, monitoring and control. Nevertheless, SDL stresses the importance of designing the learning environment in such a way that students play a role in the selection of their own learning issues and resources to achieve these goals, which  is an important feature of PBL (Loyens et al. 2008).

### Collaborative learning

Collaboration between two or more learners is known to enhance learning when learners are confronted with a complex task (Kirschner et al. 2009). Learning collaboratively is better than individual learning under certain conditions. The most important condition is the complexity of the learning task (Kirschner et al. 2009). The learning task should be ill-defined or complex, i.e. tasks with one or more unknown elements and a variety of possible solutions (Jonassen 1997). In addition, students should share a common goal and students should depend on each other’s contributions to successfully complete it; this is called interdependence theory (Torre et al. 2016).

Successful groups require sharing of knowledge and information, but also co-construction (i.e. building upon and integrating one another’s ideas), as well as having open dialogue about cognitive disagreements (Decuyper et al. 2010). Groups are complex systems in which both cognitive and social processes play a role (Van den Bossche et al. 2006). Psychological safety, i.e. a shared belief that the members of a group will not embarrass anyone for bringing forward a particular idea, is an important condition for a group to enhance learning from and with each other. Connectivity among group members, or social cohesion, also plays a role. Social cohesion might have both positive and negative effects on learning. Group members might be more committed to a task because they feel socially related, but forming too strong a social connection could be detrimental; e.g. because group members might not ask critical questions or might avoid discussing cognitive disagreements (Van den Bossche et al. 2006).

Communities of practice (CoP) introduced by Lave and Wenger (1991), is another relevant theory. The principle behind this theory is that learning involves interacting with others in a specific place and time; learning is a matter of participation in a community of members with mutual goals and interests (Cleland and Durning 2019). A community of practice contains members who learn through their participation and interactions with one another on a regular basis, during which they share practices, mutually engage, and develop around things that matter to them (Wenger 1998).

In sum, collaborative learning is beneficial for learning when students work on complex tasks, feel interdependent, have a common goal, share information and build upon each other’s contributions. In this form of learning they are assumed to discuss cognitive disagreements, feel psychologically safe and connect socially to some extent while they share practices and learn from and with each other. In PBL students also discuss complex problems in small groups in which they are expected to build upon each others’ contributions and learn from and with each other.

As explained above, instructional design theories, cognitive theories, such as prior knowledge activation theory, self-directed learning theory, and theories related to collaborative learning provide explanations of how to enhance learning. But how can we translate these principles or theories to our daily PBL practice? Part II offers some direction on how PBL practice could be aligned with the theories and principles introduced in part I.

## Part II: PBL in practice

### Contextual learning

Although learning in many PBL programs starts from tasks or problems derived from a professionally relevant context in order to stimulate transfer of learning to new situations, or is task-centered, usually it relies on written, paper-based problems including phenomena that need further explanation or a management strategy. Nowadays, other formats are used as well in PBL, such as virtual patients (Huwendiek et al. 2013), simulated patients (Tremblay et al. 2017; Behrens et al. 2018) and even real patients (Diemers et al. 2008). PBL can also be used to introduce inter-professional education; an educational intervention in which the members of more than one health profession learn interactively together (Reeves et al. 2013). Students from different disciplines, e.g. family medicine, social medicine and nursing, could discuss a problem of an elderly patient with a chronic illness in a primary care setting, visit the patient at home or in the hospital, discuss and prepare the visit in a small group, and finally discuss a care plan during the small group meeting after their visit (see https://www.youtube.com/watch?v=prKUYNGwdFI&feature=youtu.be). In this example, students are being prepared for inter-professional collaboration by confronting them with a professionally relevant problem, stimulating them to cross the boundaries of their own discipline, while engaging in an authentic context around a complex ill-defined task.

### Constructive learning

Another option to strengthen PBL is to incorporate structured peer feedback used in PBL (Dolmans et al. 2015). All too often the superficiality of discussions in PBL groups impedes deep learning. In team-based learning (TBL), team members evaluate each other’s contributions to the success of the group (Burgess et al. 2017). Although peer feedback on cognitive, social and motivational contributions has been applied in PBL, it is not used commonly yet, whereas it is known to effectively improve for students who initially make poor contributions under certain conditions (Kamp et al. 2013). Other attempts applied successfully in the past include splitting the PBL group in small study teams of three to four students who collaborate on self-study to prepare a concept map to be discussed in the next tutorial group session (Moust and Roebertsen 2010). A concept map is a schema of a set of related concepts. Concept maps are known to stimulate meaningful learning in students and enable teachers to provide feedback (Daley and Torre 2010). The various study teams present their concept map and can compare, build upon and critically evaluate each other’s concept maps. Concept maps are aimed at stimulating students to actively construct their knowledge networks, although their effectiveness might be dependent on the context in which they are used; e.g. on the degree to which students are stimulated to actively reconstruct and retrieve their knowledge (Karpicke and Blunt 2011).

### Self-directed learning

In PBL, students generate learning issues that need further self-study. They are given much time for self-study, may choose their own study approach, and may select their own learning resources. These aspects fit with the principles of SDL. Usually in PBL students are offered limited opportunities to choose learning tasks that fit their personal needs. In general, personalized education has received limited attention so far, whereas personalized curricula are regarded as a key feature of the medical school of the future (Harden 2018; Hays 2018). But, what is meant by ‘personalized curricula’? Personalized learning still lacks a widely accepted definition, but is often meant to refer to personalized instructional goals, personalized task selection, personalized instruction or levels of scaffolding and based on self-regulated learning and self-determination theory, needing further research to refine and support its’ principles (Watson and Watson 2016). Thus, in a personalized curriculum, students analyze their learning needs, set challenging goals, and select learning tasks and learning resources by themselves. With a personalized curriculum we move away from a standard ‘one-size-fits-all’ static curriculum. This is not to say that students learn individually. A particular group of students might share the same needs due to which they collaboratively can work on a particular PBL task. In such a personalized curriculum, an autonomy-supportive learning environment is offered in which students not only have some autonomy in their learning, but also feel competent and supported by and related to their teachers (Kusurkar et al. 2011; Ryan and Deci 2000). Offering students some autonomy to select learning tasks that fit their needs is expected to enhance students’ engagement and independent motivation in studying. It is also expected to enhance students’ ownership over their learning, so that they take the lead in their own learning and development. Offering autonomy does not mean, as argued above, that students no longer need support. So far, what is known about training students to become self-directed learners is that coaching and scaffolding by teachers is very much needed to facilitate self-evaluation, goal-setting and taking control of learning efforts (Sitzmann and Ely 2011). Clearly, both teachers and students need training to do so (Beckers et al. 2016). In sum, although, personalized curricula seem to fit with self-regulated learning theory and tend to provide students with autonomy to select learning tasks that fit their needs, further evidence is needed, because learners are often poor in estimating their performance and monitoring and controlling their own learning (Bjork et al. 2013). We still need to know more about how to optimally balance learner autonomy on the one hand and scaffolding on the other hand. We should offer learners support and guidance to select learning tasks and resources and need to simultaneously learn them to become better in monitoring and controlling their learning and actively reconstruct and retrieve their knowledge. How to effectively balance the two, needs further research.

### Collaborative learning

Over the years, many schools have been confronted by an increase in student numbers resulting in large cohorts that make students feel anonymous and disconnected to one another and the program. Splitting a large cohort into smaller sub-cohorts is another method being applied in PBL practice to strengthen social networks between students. Within smaller cohorts it is easier for students to connect with other students, which benefits their learning (Hommes et al. 2012, 2014). Another option is to introduce smaller cohorts of teachers who connect with smaller cohorts of students. These smaller groups of teachers who interact and share practices are called professional learning communities (PLCs) (Stoll et al. 2006). These ideas fit well with the communities of practice (CoP) theory as explained above. By intentionally building small communities of students and teachers inside a large cohort, students and teachers are expected to share practices and learn from and with each other on a regular basis. By making students and teachers feel related and connected to mutual goals of reciprocal learning and by creating student-staff partnerships we can enhance commitment and ownership and empower students and teachers to continuously improve their teaching practices (Cook-Sather et al. 2014; Bovill et al. 2016; Bendermacher et al. 2017). So teachers, should have opportunities to work collaboratively and be engaged in the (co)construction and implementation of redesigned teaching or assessment practices and set and monitor goals for themselves and their learners to enhance and improve teaching practices (Schnellert et al. 2008).

So far, nothing has been said about introducing technology in education. Technology will certainly play an important role in the school of the future (Harden 2018). Technology will be integrated in our curricula but it should serve as a vehicle that optimizes learning in line with the principles or theories stated above and not be treated as an end in itself. It can help to provide information on students’ self-assessments to support them to monitor their performance and inform teachers to adequately coach and support students, which is a first important step in helping students to become better self-directed learners, and in offering personalized curricula that fit the students’ needs.

In sum, Part II explained how theories could inform PBL practice. But, what do we know so far from PBL research? Part III deals with how theory can enhance PBL research.

## Part III: Design-based research to enhance practice and advance theory

Before discussing how PBL research can enhance practice and theory, this section begins by explaining how PBL research has evolved over time. Cook et al. (2008) developed a useful framework to classify research aims in medical education research. They distinguished descriptive, justification and clarification studies. Initially, PBL studies were merely descriptions of how to conduct PBL, accompanied by students’ and teachers’ perceptions. These studies are valuable in that they demonstrated that both students and teachers were very positive about actively involving students in PBL and as such inspired many institutions to implement PBL. After these initial positive findings, questions were raised about its effectiveness, which resulted in justification studies, which compared PBL curricula with non-PBL curricula in terms of student outcomes. These studies were also valuable in that we saw that PBL curricula have a positive effect on the acquisition of generic competencies, such as communication skills, and have a positive effect on knowledge application (Gijbels et al. 2005; Hmelo-Silver 2004; Schmidt et al. 2009; Strobel and Van Barneveld 2009). These studies led to a lot of debate due to the difficulty of comparing curricula. There are two reasons. First, educational interventions are not blinded and second, it is hard to control for contextual differences, such as the way PBL is implemented in different curricula (Norman and Schmidt 2000). Thereafter, a third wave of explanatory studies entered the field, usually qualitative studies aimed at disentangling the underlying success and hindering factors that many institutions face worldwide in daily PBL practice. These explanatory studies were very beneficial to advance our understanding of PBL in various contexts and settings (see e.g. Frambach et al. 2012, 2014). In sum, PBL research evolved from the descriptive, to justification and then clarification studies. Over the years the number of published studies has grown enormously.

To what extent has daily PBL teaching practice been inspired by these studies? It has definitely benefited and still benefits today. For instance, in the past practitioners wondered whether a tutor should first and foremost be a content expert or a process expert. Nowadays, there is clear evidence available that a good PBL tutor should be both a content and process expert (Dolmans et al. 2002). Does this imply that there are no gaps left in the current literature on PBL? I am not convinced that this is true for several reasons, even if I have to admit that it might be somewhat difficult to reflect critically on a topic I have been involved with for almost 3 decades. Why do I think we need further research?

Education is complex and involves many different variables interacting with each other (Berliner 2002). Besides that, education is highly context-specific. The research often has ambiguous and inconsistent findings (Dolmans et al. 2016). We still do not fully comprehend how and why PBL works or does not work. Design-based research seems to offer a promising way to move forward.

Many medical studies published in 2003 and 2004 were initially mainly quantitative outcome-oriented justification studies (Cook et al. 2008). The same holds for studies published on PBL till mid 2000 (Woei et al. 2019). More clarification studies are needed to gain a deeper understanding (Cook et al. 2008). Given that context matters in learning, DBR studies are conducted in naturalistic settings and not in the laboratory, where variables are investigated in isolation (Barab and Squire 2004). In the natural learning environment, DBR studies (re)design the learning context, based on theory, in collaboration with stakeholders (teachers, students, educational designers, researchers), using mixed methods to investigate the variables in iterative cycles of analysis, design, and evaluation (Brown 1992; Collins 1992; Barab and Squire 2004; McKenney and Reeves 2013; Bakker 2018; Dolmans and Tigelaar 2012).

DBR aims to improve practice through the strong involvement of all stakeholders in the analysis, design and evaluation phase. It also aims to advance our understanding, given its focus on building and testing theory. In other words, building or testing theory and practice improvements are intertwined processes in DBR, requiring both researchers and practitioners to collaborate and interact closely.

Typically, DBR studies try to answer a research question such as: How can PBL with certain characteristics better enable students to achieve a particular outcome? The particular characteristics are where theory plays a role (Bakker 2018). Theory provides the guidelines or principles for designing a PBL intervention. For example, would splitting a PBL practice involving a large cohort of students and teachers in smaller sub-cohorts better enable students to take a deep approach to learning in a third-year health sciences program? Implementing and investigating this ‘redesigned’ PBL approach to teaching practice allows researchers to collect evidence for why a PBL intervention with particular characteristics might work in a particular context or not work. The theory chosen in this DBR example derives from the CoP theory mentioned above. Usually not one theory but a mixture of theories or guidelines will be combined to design (or redesign) and investigate the intervention. The theory will inform the design of the intervention and the methods and instruments used. In addition, it is important to keep in mind that DBR research often consists of a series of studies (Bakker 2018); i.e. the research question is broad and will usually be split in sub-questions, such as, what is an appropriate PBL design, how well is the PBL design implemented, and what are its effects?

Which research questions and themes could be relevant for DBR to address? First, PBL studies usually focus on PBL in classroom settings, whereas far more research is needed on PBL as a method to link classroom and workplace learning. PBL research has mainly focused on single professional learning environments, whereas far more research is needed on PBL in inter-professional learning environments. Previously, PBL research focused on cognitive aspects of learning and has only recently begun addressing the social aspects of learning. PBL research in blended or technology-enhanced learning environments is also scarce and needs further research (De Jong et al. 2017; Verstegen et al. 2018). To date, PBL studies usually look at standard ‘one size fits all’ learning formats due to which not much is known about personalized curricula that fit the students’ PBL needs.

## Conclusion and discussion

PBL should continually be adapted to align it better with such principles as contextual, constructive, self-directed and collaborative learning and by embracing effectively seminal and new theories. We should not stick to a one-size fits all PBL approach, but adapt and redesign PBL and other approaches based on theories.

Do such adaptations represent hybrid PBL solutions? As long as they stimulate students to take a deep approach to learning and fit the four principles—contextual, constructive, self-directed, and collaborative learning—they can be called PBL innovations. Violations against any one of these principles may be seen as moving a step away from the initial aims behind PBL.

Do DBR studies test and build universal theory or are DBR findings just locally relevant? Given that DBR studies are conducted in the real environment, they are locally relevant and provide useful insights for practice. However, because the intervention or design is connected to theoretical claims that go beyond the local context (Barab and Squire 2004), they also offer a promising approach to advance our theoretical understanding, although more research is needed to demonstrate that it actually does advance both theory and practice (Anderson and Shattuck 2012). Theory alone will not solve all the problems encountered in practice (Cleland and Durning 2019), but it can help explain how and why problems could be solved and help us advance our understanding of how PBL with particular characteristics may facilitate students to achieve better results.

Finally, we should keep in mind that there is no one-size-fits-all solution to address the problems encountered in PBL. We should be very careful in drawing conclusions about which PBL approach works best. No single solution works optimally under all conditions. At most, DBR can help us gain better insight into why PBL with certain characteristics, preferably based on theory, might work in a specific context with particular goals in mind. The theory can help us explain how complex phenomena in PBL interact and thereby add to our understanding. PBL needs to be continually redesigned in line with current and newly emerging theories. PBL is highly context-specific and needs to be reinvented again and again given that its context is not fixed, but changes continually.
